# The efficacy of contralaterally controlled functional electrical stimulation compared to conventional neuromuscular electrical stimulation for recovery of limb function following a stroke: a systematic review and meta-analysis

**DOI:** 10.3389/fneur.2024.1340248

**Published:** 2024-02-21

**Authors:** Alhussain Halawani, Ammar Aljabri, Dena M. Bahathiq, Roaa E. Morya, Saeed Alghamdi, Seraj Makkawi

**Affiliations:** ^1^College of Medicine, King Saud Bin Abdulaziz University for Health Sciences, Jeddah, Saudi Arabia; ^2^King Abdullah International Medical Research Center, Jeddah, Saudi Arabia; ^3^Neuroscience Department, King Faisal Specialist Hospital and Research Center, Jeddah, Saudi Arabia; ^4^Department of Neuroscience, Ministry of National Guard-Health Affairs, Jeddah, Saudi Arabia

**Keywords:** neuromuscular electrical stimulation, stroke, limb paresis, rehabilitation, contralaterally controlled functional electrical stimulation

## Abstract

**Introduction:**

Limb paresis following a stroke is a common sequela that can impact patients’ quality of life. Many rehabilitation strategies targeting the restoration of motor function exist. This systematic review and meta-analysis aim to evaluate the effects of contralaterally controlled functional electrical stimulation (CCFES) as a modality for limb rehabilitation. Unlike conventional neuromuscular electrical simulation (NMES), the contra-laterality in CCFES is achieved by two methods a bend angle sensor or an electromyographic bridge (EMGB) method, both of which targets signals from the unaffected limb.

**Method:**

This review study was performed following the preferred reporting item for systematic review and meta-analysis (PRISMA) guidelines. Records that met the inclusion criteria were extracted from the following databases: Medline, Embase, and Cochrane Register of Controlled Trials (CENTRAL). Additional articles were also retrieved from clinicaltrials.gov and China/Asia on Demand (CAOD). Only randomized controlled studies (RCTs) were included.

**Results:**

Sixteen RCTs met the inclusion criteria, and 14 of which were included in the quantitative analysis (meta-analysis). The results of the analysis show that when compared to conventional NMES, CCFES displayed a better improvement in the upper extremity Fugl–Meyer assessment (UEFMA) (SMD = 0.41, 95% CI: 0.21, 0.62, *p*-value <0.0001, *I*^2^ = 15%, GRADE: moderate), box and blocks test (BBT) (SMD = 0.48, 95% CI: 0.10, 0.86, *p*-value = 0.01, *I*^2^ = 0%, GRADE: very low), modified Barthel index (mBI) (SMD = 0.44, 95% CI: 0.16, 0.71, *p*-value = 0.002, *I*^2^ = 0%, GRADE: moderate), active range of motion (AROM) (SMD = 0.61, 95% CI: 0.29, 0.94, *p*-value = 0.0002, *I*^2^ = 23%, GRADE: moderate), and surface electromyography (sEMG) scores (SMD = 0.52, 95% CI: 0.14, 0.90, *p*-value = 0.008, *I*^2^ = 0%, GRADE: low). The results of the subgroup analysis for the type of sensor used in CCFES shows that an EMGB (SMD = 0.58, 95% CI: 0.33, 0.84, *p*-value <0.00001, *I*^2^ = 7%) is more effective than a bend angle sensor (SMD = 0.17, 95% CI: −0.12, 0.45, *p*-value = 0.25, *I*^2^ = 0%).

**Conclusion:**

The results of this study provide strong evidence that shows CCFES being a better electrical stimulation modality compared to conventional NMES. This could be explained by the fact that CCFES is bilateral in nature which offers a platform for better neuroplasticity following a stroke. There is still a need for high-quality studies with a standardized approach comparing CCFES to other treatment modalities.

**Systematic review registration:**

https://www.crd.york.ac.uk/prospero/display_record.php?RecordID=342670, identifier CRD42022342670.

## 1 Introduction

A stroke is defined as a cerebrovascular accident in which sudden death of brain cells occurs as a result of vascular insufficiency, leading to poor brain perfusion and, ultimately, neurological deficits. Pathologically, stroke can be classified as either ischemic or hemorrhagic, whereupon the former accounts for roughly 85% of cases (1). Stroke is the second-leading cause of death after ischemic heart disease, and in 2019, there were approximately 12.2 million incidents and 6.55 million deaths attributed to stroke (2). Complications following a stroke range from late medical to musculoskeletal and psychosocial sequelae. The most predominant of which is upper limb impairment, which occurs in 80% of stroke survivors (3, 4). Hemiparesis ensues as a result of a defect in the signal transmission all the way from the motor cortex to the spinal cord and the corresponding muscles, resulting in an inability to move the affected limb. Furthermore, it may hinder an individual’s functionality and independence in performing activities of daily living (ADLs) (5).

Many rehabilitation strategies exist that aim to restore the motor function of the paretic limb. These include occupational therapy, physiotherapy, constraint-induced movement therapy, and mirror therapy. Additionally, recent advances in rehabilitation include robotic-aided therapy and impairment-oriented training (4). While these approaches can be beneficial to some extent, the evidence regarding their overall efficacy is still quite controversial. Moreover, these strategies lack standardization and are impractical as they are difficult to administer. Thus, there is still a need for newer forms of therapies for limb impairment (4, 6).

Conventional neuromuscular electrical stimulation (NMES) is a good alternative. Recent studies have shown that conventional NMES significantly improves upper limb motor function when compared to another form of therapy (7–9). Conventional NMES devices induce muscular contraction in the paretic limb by using an electrical current that stimulates the lower motor neurons. The current is passed by surface electrodes that are attached to the motor points of the paretic muscles (10). Common modalities used in conventional NMES are cyclic NMES and electromyographic (EMG)-triggered NMES. In cyclic NMES, stimulations occur in an on and off cycle as it is set up by the operator. However, in EMG-triggered NMES, the stimulation concurs with the patient’s effort to move the paretic limb. Once the pre-set threshold of the surface EMG signal is reached, an electrical current is triggered, aiding the movement of the limb (10).

Contralaterally controlled functional electrical stimulation (CCFES) is a novel form of electrical stimulation therapy, introduced first by Knutson et al. (11). Similarly, to NMES, CCFES involves electrical stimulation to the neuromuscular system, however, the contralateral nature of the electrical signals is what makes it distinctive from the other modalities as it uses the unaffected limb movements to induce an electrical current in the weakened limb (10). The way in which the device works is by having the subjects wear a glove with a bend angle sensor on their normally functioning hand. The magnitude and intensity of the stimulation are governed by the degree of movement from their glove-worn hands (11). Similar to Knutson’s methods, Zhou et al. (12) use the healthy muscles from the unaffected limb to stimulate the paretic limb. However, instead of a bend angle sensor, multiple EMG sensors in the muscles of the non-paretic limb are bridged to its corresponding muscles in the affected limb, allowing for multi-movement training (12).

The sensorimotor cortex in the brain is not static, but in fact, modifiable through different sensory and motor inputs (13). This neuroplasticity principle provides the bases for electrical rehabilitation since they allow for synchronous repetitive movements, which in turn boost the remodeling of synapses and the organization of neurons, resulting in improved motor functioning (6). An additional advantage CCFES therapy has over conventional NMES is that it provides bilateral symmetrical movements. Studies have observed significant cortical modulation following bilateral arm training compared to unilateral training (14, 15).

Previous systematic reviews have explored the effects of CCFES compared to conventional NMES (16, 17). A meta-analysis by Loh et al. (16) found that CCFES significantly improved motor function compared to conventional NMES. However, the study was limited by the low number of randomized control trials (RCTs). Hendawy et al. (17), on the other hand, report that the evidence regarding the efficacy of CCFES was insufficient. The aim of our study is to conduct a systematic review and meta-analysis to expand upon the work of these previous reviews by incorporating additional RCTs as well as conducting a subgroup analysis that measures the effects of the interventions on the different stroke phases, the long-term effects, and the nature of the contralateral sensory used.

## 2 Methods

This review was conducted in accordance with the preferred reporting item for systematic reviews and meta-analysis (PRISMA) guidelines (18). The guidelines can be found in Supplementary Appendix 1. The prespecified protocol was registered on the 30th of June and accepted on the 8th of July 2022 with the International Prospective Register of Systematic Reviews (PROSPERO), registration number: CRD42022342670.

### 2.1 Eligibility criteria

In this review, RCTs that evaluated the efficacy of CCFES when compared to conventional NMES or any other forms of rehabilitation were included. The study population was adult stroke survivors (age >18) with limb paresis, either in the upper or lower limbs. All phases of stroke were included; acute, subacute, and chronic. The outcome measures assessed in this review are related to motor function and activity level assessment. The population, intervention, control, and outcomes (PICO) model for this review can be found in Box 1. The search was limited to English and Chinese studies; any other languages were excluded from this review. Additionally, abstracts, trials with unpublished results, inaccessible studies, and non-RCTs designs were all excluded as well.

BOX 1 **PICO model.**

**Table:** 

**Participants**
► Stroke survivors with paresis either in lower or upper limb ► Adults (age more than 18) ► All stroke phases (acute, subacute, chronic)
**Intervention**
► Contralaterally controlled functional electrical stimulation ► Electromyographic Bridge therapy ► Bend Angle Sensor ► Any other modalities that achieves bilateral electrical stimulation
**Comparisons**
► Neuromuscular electrical stimulation; either cyclic or EMG-triggered ► Any other forms of rehabilitation.
**Outcome measures**
► Primary Outcome: ► Motor functional assessment (UEFMA, LEFMA, BBT, AROM) ► Activities of daily living assessment (mBI, AMAT, ARAT) ► Secondary outcome: (sEMG ratio)

EMG, electromyographic; UEFMA, upper extremity Fugl–Meyer assessment; LEFMA, lower extremity Fugl–Meyer assessment; BBT, box and blocks test; AROM, active range of motion; mBI, modified Barthel index; AMAT, arm motor ability test; ARAT, action research arm test; sEMG, surface electromyography.

### 2.2 Search strategy and the selection process

On the 2nd of June 2022, the following databases were systematically searched: Medline, Embase, and Cochrane Register of Controlled Trials (CENTRAL) since their inception. The search strategy for the databases can be found in Supplementary Appendix 2. Moreover, the US National Institutes of Health Clinical Trials Registry^1^ was also searched, as well as the website China/Asia on Demand (CAOD: https://caod.oriprobe.com). Furthermore, a manual search of the references identified by the systematic search was also conducted for any additional articles.

The references identified from the search strategy were imported to Covidence software, a web-based tool (19), in which duplicates were automatically removed. The screening process was carried out by four independent authors (AH, AA, DB, and RM) and was done in two steps. First, a title and abstract screening. Second, a full-text screening for eligibility assessment. Any disagreements between the authors were resolved by consensus. Consultation of a third party was not required.

### 2.3 Data extraction

The data extraction process was done by four authors (AH, AA, DB, and RM) in duplicates through a prepared data collection sheet. The extracted variables from each of the included studies were as follows: study design, number of participants, baseline characteristics of participants and the stroke phase, the maximum follow-up duration, the content and duration of therapy, the stimulated muscle/s, the nature of the contralateral sensor, and all the outcome measures assessed.

### 2.4 Outcomes

The primary outcomes for this review were classified according to the International Classification of Functioning, Disability, and Health (ICF) (20) as either body functions and structures or activity level assessment. Body functional assessment outcomes include the Fugl–Meyer assessment (FMA), box and blocks test (BBT), and the active range of motion (AROM). The activity level assessment outcomes include arm motor ability test (AMAT), Barthel index (BI), and the action research arm test (ARAT).

The FMA scale contains 226 points distributed across five domains: motor and sensory function, balance, joint pain, and range of motion. Each item in each domain is scored as either 0, 1, or 2, where 0 equals no performance, 1 equals partial performance, and 2 equals full performance. The motor domain ranges from 0 (hemiplegic) to 100 (normal motor function) and is divided into 66 points for the upper extremity Fugl–Meyer assessment (UEFMA) and 34 points for the lower extremity Fugl–Meyer assessment (LEFMA) (21).

BBT measures gross manual dexterity. It involves the patient carrying as many wooden blocks over a partitioned box and releasing it in a span of 60 s. The more blocks transferred, the higher the score (22).

AMAT is used to measure the range of ADLs. The test is comprised of 13 compound ADLs tasks, and each task is rated according to two 6-step rating scales. The first is the functional ability scale, at which a 0 score equals no use, and a 5 score equals normal use. The second is the quality of movement scale, where a 0 score equals no movement initiation, and a 5 score equals normal movement (23).

The BI is another test that measures ADLs performance, and it contains 10 different ADLs tasks. Another version of the test exists, called the modified Barthel index (mBI). The only distinguishing feature between the two is that the modified version is measured at a 5-point functional ability scale instead of a 3-point scale (22).

The ARAT measures upper extremity functional limitation through 19 arm motor tests which are spread into 4 subsets: grasp, grip, pinch, and gross motor. Each test is rated on a 4-point scale where 0 equals unable, and 3 equals normal. The total score is 57, at which a higher score indicates a lesser degree of impairment (24).

The secondary outcome was surface electromyography (sEMG), which measures muscle activity. The data was reported as the root mean square (RMS) as it standardizes individual differences, such as total body fat and weight (25).

### 2.5 Risk of bias assessment

The risk of bias was assessed using the revised Cochrane Risk of Bias 2 (RoB 2) tool (26) and was done by four authors (AH, AA, DB, and RM) in duplicates. Any disagreements between the authors were resolved by consensus. The tool is designed to assess the risk of bias for randomized trials through a series of signaling questions. Five domains are assessed, and a judgment of low, some concern, or high risk is generated by an algorithm based on the answers to the signaling questions. An overall risk of bias is then given depending on the judgment of each domain. The risk of bias summary and graph images were generated using the robvis web visualization tool (27).

### 2.6 Meta-analysis

The meta-analysis was conducted using Review Manager (RevMan5) version 5.4.1 software (Cochrane Collaboration) (28). Studies that compared CCFES to conventional NMES were included in the quantitative synthesis. Studies in which the control group received rehabilitation other than conventional NMES were narratively described in the qualitative synthesis and were not included in the meta-analysis. Analysis was performed when two or more studies’ data could be combined in accordance with each outcome measure. A random-effects model was used, and all outcomes were pooled by the inverse variance weighting method. Data with different scales in each of the continuous outcomes were converted to the same scale using the Standardized mean difference (SMD), and the confidence interval (CI) was set at 95%. A *p*-value <0.05 was considered significant. The *I*^2^ statistic, along with the chi-squared test, were used to test for statistical heterogeneity. An *I*^2^ value greater than 50% was considered to represent significant heterogeneity, in which case a sensitivity analysis was performed. A subgroup analysis in regard to the follow-up and type of sensor used was carried out for the FMA outcome. For all the other outcomes, the data for the furthest follow-up were used in the analysis. If an outcome measure was reported in at least 10 studies, then a funnel plot was generated to be examined visually for any asymmetry which would indicate the presence of a publication bias.

### 2.7 Certainty of evidence

The quality of evidence for each outcome was appraised using the Grading of Recommendations Assessment, Development and Evaluation (GRADE) criteria. For each outcome measure, a rating of either high, moderate, low, or very low was given depending on GRADE’s approach for rating the body of evidence. Study design, risk of bias, inconsistency, indirectness, and imprecision are some of the factors upon which the certainty of the evidence was appraised (29).

## 3 Results

### 3.1 Study selection

**Figure 1:**
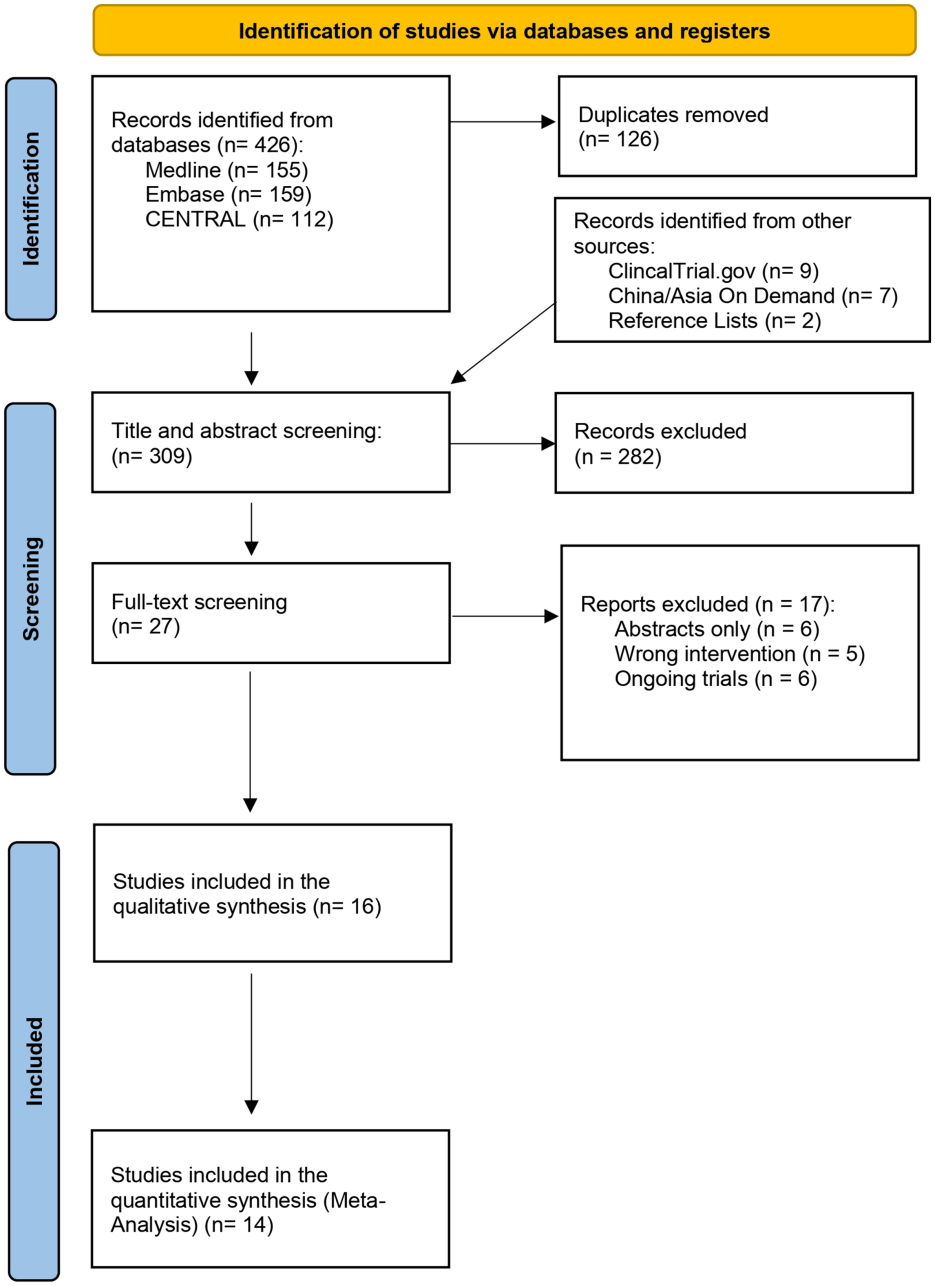
Preferred reporting items for systematic reviews and meta-analyses (PRISMA) flow diagram.

The electronic search initially yielded 426 articles, and after duplicates were removed, 309 articles were screened for relevance by their titles and abstracts. Additional nine articles have been identified from other sources and were included and assessed for relevancy. A total of 36 articles were sought for retrieval and received a full-text screening for eligibility. Of the 36 articles, 16 articles met the inclusion criteria and were included in the qualitative syntheses (30–45). Within the included articles, two did not meet the criteria for the quantitative synthesis (meta-analysis) and were narratively described (44, 45). The details of the selection process can be viewed in the PRISMA flowchart, Figure 1.

### 3.2 Characteristics of included studies

A total of 570 participants were included in this review, of which 540 (273 in the CCFES group and 267 in the conventional NMES group) were included in the meta-analysis. Two studies included in our analysis, Knutson et al. (32) and Yang et al. (41), have allocated participants into three groups. In regard to Knutson et al., the three groups were: arm + hand CCFES, hand CCFES, and arm + hand NMES. For Yang et al., the groups were: CCFES, intensive CCFES, and conventional NMES. In our analysis, however, the hand CCFES and the intensive CCFES group were not included. Regarding the nature of the contralateral sensor, six studies have used a bend angle sensor (30–32, 35, 36, 43), and nine studies used an EMGB (33, 34, 37–42, 44). For the classification of stroke phases, participants within the first 2 weeks post-stroke were regarded as acute, 3–24 weeks were subacute, and more than 24 weeks were chronic (46). Therefore, one study assessed participants in the acute phase (40), one in the acute/subacute (34), nine studies investigated subacute participants (30, 33, 35–39, 41, 42), one in the subacute/chronic (32), and finally, four studies examined chronic individuals (31, 43–45). The duration of therapy differed between the included studies. The therapy program’s duration ranged from 2 weeks (40), 3 weeks (33, 35, 36, 41, 42), 4 weeks (34, 37, 39, 41, 44) 6 weeks (30, 43), and 12 weeks (31, 32). Six studies (30–32, 38, 43, 45) followed up with the participants after the treatment, with the follow-up period ranging from 1–6 months. Only two studies assessed the intervention on the lower limbs (42, 43). The main characteristics of the included studies are summarized in Table 1.

**Table 1:** Characteristics of included studies.

Author, year	Study design	Number of participants		Mean age (standard deviation)		Stroke phase	Maximum follow-up period	Content of therapy	Duration of therapy	Contralateral sensor	Outcome measures
		Total	Intervention (control)	Intervention	Control						
Knutson, 2012	Randomized control trial (RCT)	21	10 (11)	54.4 (13.5)	51.9 (7.9)	Subacute	3 months	A. Therapist-guided FTP B. Self-administered homebased electrical stimulations	Total: 6 weeks, 12 h/week A. 2 × 90 min session/week B. 2 session/day, 75 min/session	Bend angle sensor	AROM, BBT, UEFMA, AMAT, tracking error
Knutson, 2016	Randomized control trial (RCT)	80	40 (40)	55.4 (17.0)^*^	56.3 (12.7)^*^	Chronic	6 months	A. Therapist-guided FTP B. Self-administered homebased electrical stimulations	Total: 12 weeks, 10 h/week A. 2 × 60 min session/week B. 10 × 50 min session/week	Bend angle sensor	BBT, UEFMA, AMAT
Knutson, 2020	Randomized control trial (RCT)	67	28 (11)	54 (12.6)	61 (12.5)	Subacute/chronic	6 months	A. Therapist-guided FTP B. Self-administered homebased electrical stimulations	Total: 12 weeks, 10 h/week A. 2 × 70 min session/week B. 10 × 46 min session/week	Bend angle sensor	BBT, UEFMA, SULCUS, AMAT, reachable workspace
Shen, 2015	Randomized control trial (RCT)	60	30 (30)	59.7 (15.2)	60.9 (13.5)	Subacute	Nil	A. Therapist-guided wrist extension	Total: 3 weeks A. 5 × 20 min session/week	Electromyographic Bridge	UEFMA, AROM, MI, FTHUE-HK
Huang, 2017	Randomized control trial (RCT)	48	24 (24)	55.0 (8.85)	53.7 (11.72)	Acute/subacute	Nil	A. Therapist-guided wrist extension	Total 4 weeks A. 6 × 20 min sessions/week	Electromyographic bridge	UEFMA. WMFT, MBI
Huang, 2018	Randomized control trial (RCT)	32	16 (16)	56.18 (13.17)	62.37 (12.54)	Subacute	Nil	A. Therapist-guided wrist extension B. Routine Rehabilitation	Total 3 weeks A. 5 × 20 min sessions/week B. 5 × 1 h a day/week	Bend angle sensor	UEFMA, ARAT, BI, RMS
Huang, 2021	Randomized control trial (RCT)	50	25 (25)	56.2 (12.2)	60.4 (11.3)	Subacute	Nil	A. Therapist-guided wrist extension B. Routine rehabilitation	Total: 3 weeks A. 5 × 20 min sessions/week B. 5 × 1 h a day/week	Bend angle sensor	UEFMA, ARAT, BI, RMS
Zhao, 2021	Randomized control trial (RCT)	44	22 (22)	58.90 (8.52)	56.82 (7.34)	Subacute	Nil	A. Therapist-guided shoulder flexion and abduction	Total: 4 weeks A. 5 × 20 min sessions/week	Electromyographic bridge	EMG, AROM, UEFMA
Zhao, 2022	Randomized control trial (RCT)	24	16 (8)	52.75 (17.16)	53.88 (10.70)	Subacute	1 month	A. Therapist-guided wrist extension	Total: 4 weeks, 40 sessions A. 2 × 10 min sessions/day for 5 days/week	Electromyographic bridge	AROM, FMA-UE, MMT, BI
Zhou, 2017	Randomized control trial (RCT)	42	22 (20)	50.9 (13.8)	56.9 (10)	Subacute	Nil	A. Therapist-guided wrist extension B. Physical therapy C. Occupational therapy	Total: 4 weeks. A. 2 × 25 min session/week B. 5 × 40 min session/week C. 5 × 40 min session/week	Electromyographic bridge	Brunnstrom’s stages, UE-FMA, MSS, sEMG ratio, AROM (wrist/finger extension)
Zheng, 2019	Randomized control trial (RCT)	50	25 (25)	63.38 (12.14)	61.35 (12.13)	Acute	Nil	A. Therapist-guided wrist extension	Total: 2 weeks. A. 5 × 40 min session/week	Electromyographic bridge	UEFMA, AROM (wrist dorsiflexion), MMT (extensor carpi), JHFT, mBI
Yang, 2021	Randomized control trial (RCT)	45	14 (15)	55.22 (11.34)	57.37 (12.17)	Subacute	Nil	A. Therapist-guided wrist extension B. Physical therapy C. Occupational therapy	Total: 3 weeks. A. 5 × 20 min session/week B. 5 × 30 min session/week C. 5 × 30 min session/week	Electromyographic bridge	UEFMA, MBI, AROM, sEMG
Shen, 2022	Randomized control trial (RCT)	44	22 (22)	62.86 (12.96)	66.09 (6.38)	Subacute	Nil	A. Therapist-guided ankle dorsiflexion B. Routine rehabilitation	Total: 3 weeks A. 5 × 15 min session/week B. N/A	Electromyographic bridge	LEFMA, MBI, sEMG, aEMG, iEMG, RMS
Knutson, 2013	Randomized control trial (RCT)	26	14 (12)	56.7 (13.7)	59.3 (9.1)	Chronic	3 months	A. Therapist-guided electrical stimulation and gait training B. Self-administered homebased electrical stimulations	Total: 6 weeks A. 2 × 45 min session/week B. 10 × 51 min session/week	Bend angle sensor	LEFMA, AROM, maximum dorsiflexion movement, maximum dorsiflexion angle, gait velocity, stride length, cadence, time to complete mEFAP, peak knee & hip flexion during swing
Kim, 2014	Randomized control trial (RCT)	33	11 (11)	58.10 (8.32)	62.11 (1.37)	Chronic	Nil	A. Therapist-guided wrist extension + mirror therapy B. Conventional physical therapy	Total: 4 weeks A. 5 × 30 min session/week B. 5 × 30 min session/week	Electromyographic bridge	MMT, AROM, MAS, BBT, SSQOL, FIM, JTHT
Carda, 2017	Randomized control trial (RCT) (cross-over design)	11	5 (6)	45.6 (14.5)	49.8 (13.3)	Chronic	4.5 months	A. Therapist-guided EAMT B. Goal-oriented occupational therapy	A. 10 × 90 min session/day for 2 weeks cross-over B. 10 × 90 min session/day for 2 weeks	N/A	UEFMA, WMFT time, WMFT-FAS, REPAS, MAL-AOU, MAL-QOM

^*^Data is represented as median (interquartile range). UEFMA, upper extremity Fugl–Meyer assessment; MBI, modified Barthel index; AROM, active range of motion; BBT, box and blocks test; AMAT, arm motor ability test; SULCUS, stroke upper limb capacity scale; WMFT, Wolf motor function test; BI, Barthel index; JHFT, Jebsen hand function test; MMT, manual muscle testing; SSQOL, stroke specific quality of life scale; FIM, functional independence measure; REPAS, resistance to passive movement scale; MAL-AOU, motor activity log amount of use; MAL-QOM, motor activity log quality of movement; sEMG, surface electromyography; aEMG, average electromyography; iEMG, integrated electromyography; RMS, root mean square; JTHT, Jebsen Taylor hand test; mEFAP, modified Emory functional ambulation profile; MI, motricity index; MAS, modified Ashworth scale; ARAT, action research arm test; MSS, motor status scale; FTHUE-HK, Hong Kong version of functional test for the hemiplegic upper extremity; LEFMA, lower extremity Fugl–Meyer assessment; FTP, functional task practice; CCFES, contralaterally controlled functional electrical stimulation; NMES, neuromuscular electrical stimulation.

### 3.3 Risk of bias in the studies

**Figure 2:**
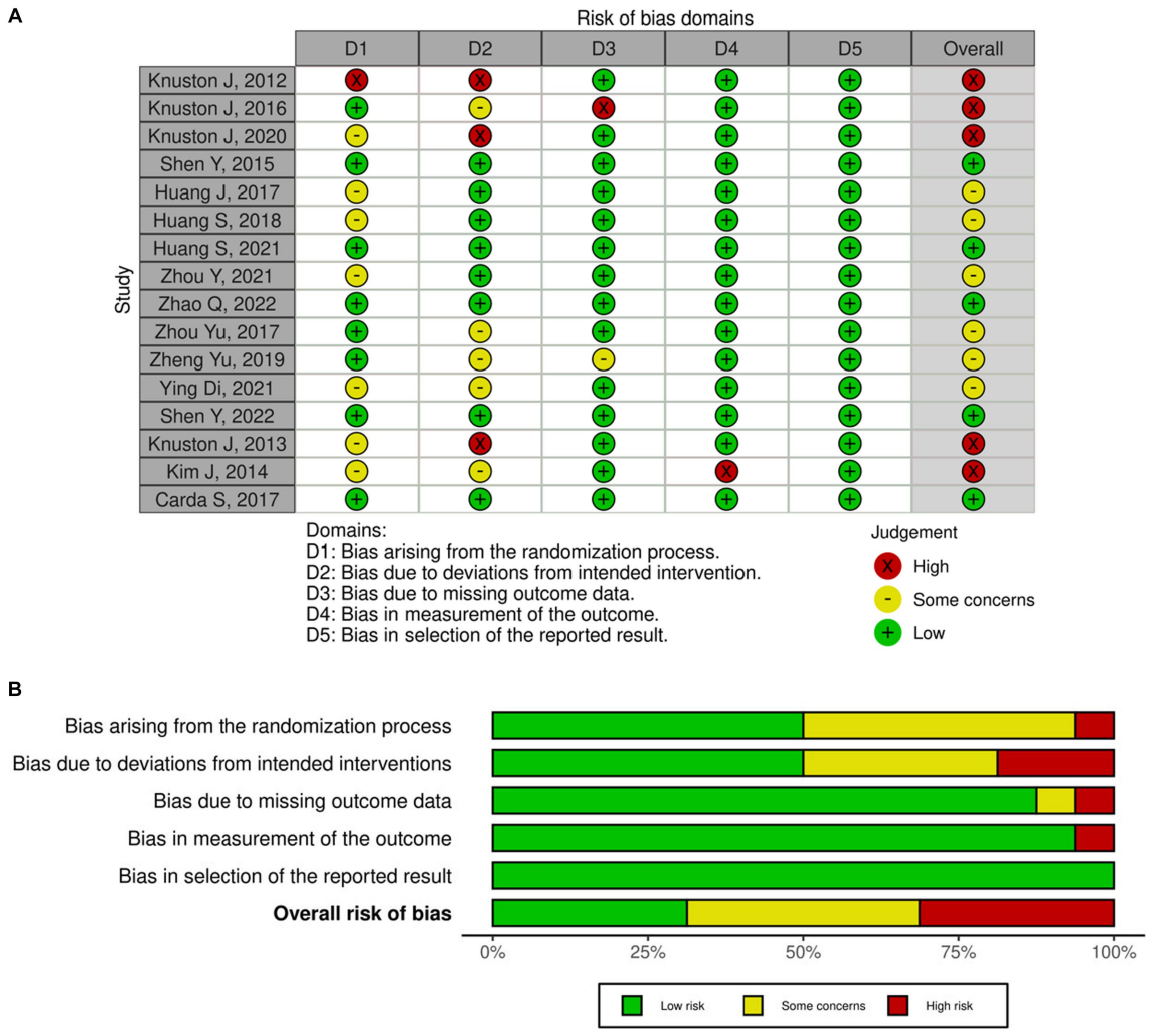
**(A)** Risk of bias summary for each study. **(B)** Risk of bias summary.

Upon assessing the risk of bias using the revised Cochrane RoB 2 tool, five studies demonstrated a high risk of bias, six studies had some concerns, and five studies appeared to have a low risk of bias. One study exhibited a risk of bias for domains 1 and 2. Two studies showed high risk in domain 2. Moreover, two additional studies denoted a high risk of bias in domains 3 and 4, respectively. Although none of the included studies blinded the participants nor the care administrators, it is unlikely for deviations from intended interventions to have occurred. However, for studies that issued a self-administered home-based intervention, deviations could have occurred, and thus they were assessed to have a high risk of bias in domain 2. Figures 2A,B provides the risk of bias graph and summary for all the included studies.

### 3.4 The efficacy of the interventions

#### 3.4.1 Upper extremity Fugl–Meyer assessment

**Figure 3:**
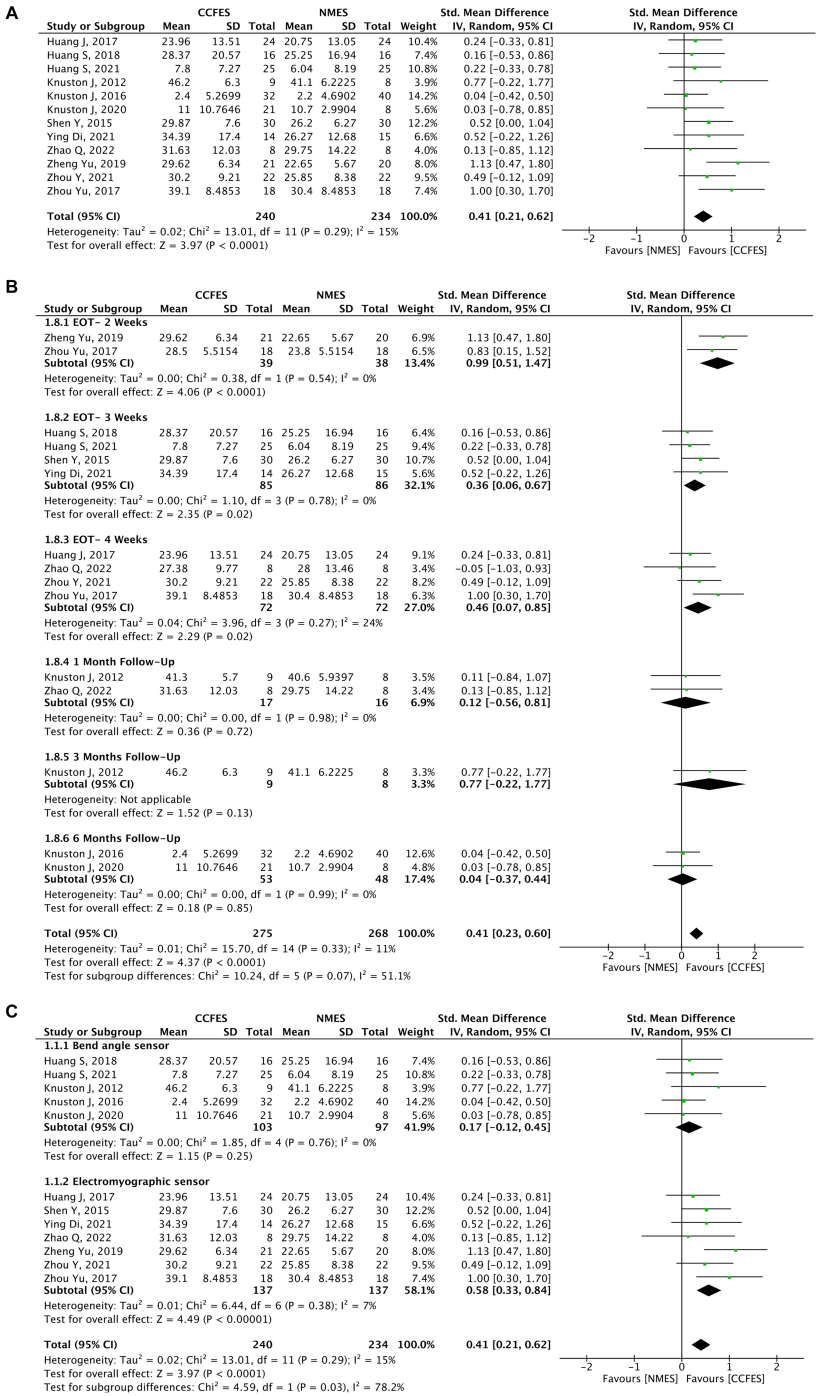
**(A)** Forest plot for upper extremity Fugl–Meyer assessment (UEFMA). CCFES, contralaterally controlled functional electrical stimulation; NMES, neuromuscular electrical stimulation; SD, standard deviation; CI, confidence interval; Std. mean difference, standardized mean difference. **(B)** Forest plot of upper extremity Fugl–Meyer assessment (UEFMA) at different follow-up periods. CCFES, contralaterally controlled functional electrical stimulation; NMES, neuromuscular electrical stimulation; SD, standard deviation; CI, confidence interval; Std. mean difference, standardized mean difference. **(C)** Subgroup analysis of upper extremity Fugl-Meyer assessment (UEFMA) type of sensor used. CCFES, contralaterally controlled functional electrical stimulation; NMES, neuromuscular electrical stimulation; SD, standard deviation; CI, confidence interval; Std. mean difference, standardized mean difference.

Comparison of the effects of CCFES with conventional NMES based on the UEFMA was measured in 13 studies (30–41, 45), of which 12 (*N* = 474) were included in the pooled analysis. Carda et al. (45) compared CCFES to a non-conventional NMES therapy, and thus, it was excluded. The results obtained from the analysis show significant improvement in the UEFMA in favor of the CCFES group (SMD = 0.41, 95% CI: 0.21, 0.62, *p*-value <0.0001, *I*^2^ = 15%, GRADE: moderate). Forest plot is shown in Figure 3A. Supplementary Appendix 3 shows the GRADE score for this outcome and the subsequent outcomes. Publication biased on the funnel plot showed no asymmetry, refer to Supplementary Appendix 4.

A subgroup analysis was performed comparing the effects of the intervention at different assessment periods, which suggests that end-of-treatment assessment (2, 3, 4 weeks) results were statistically significant compared to different follow-up periods (1, 3, 6 months) (Figure 3B).

Another subgroup comparing the type of sensor used, shows that an electromyographic sensor is more effective (SMD = 0.58, 95% CI: 0.33, 0.84, *p*-value <0.00001, *I*^2^ = 7%) than a bend angle sensor (SMD = 0.17, 95% CI: −0.12, 0.45, *p*-value = 0.25, *I*^2^ = 0%) (Figure 3C).

In Carda et al. (45), electrical assisted movement therapy (EAMT) was compared to occupational therapy in a cross-over design where the former was found to be significantly better (Mann–Whitney, *U* = 3.00, *p* < 0.05).

#### 3.4.2 Lower extremity Fugl–Meyer assessment

**Figure 4:**
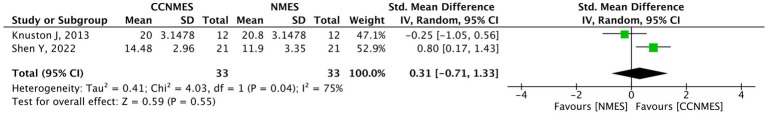
Forest plot of lower extremity Fugl–Meyer assessment (LEFMA). CCFES, contralaterally controlled functional electrical stimulation; NMES, neuromuscular electrical stimulation; SD, standard deviation; CI, confidence interval; Std. mean difference, standardized mean difference.

Only two studies (42, 43), with a total sample size of 66, explored the effects of CCFES and conventional NMES on the lower limbs. The results of the meta-analysis show no significant difference between the two groups (SMD = 0.31, 95% CI: −0.71, 1.33, *p*-value = 0.55, *I*^2^ = 75%, GRADE: very low). The results display a significant heterogeneity for which a sensitivity analysis could not be performed due to the low number of studies. The forest plot is depicted in Figure 4.

#### 3.4.3 Active range of motion

**Figure 5:**
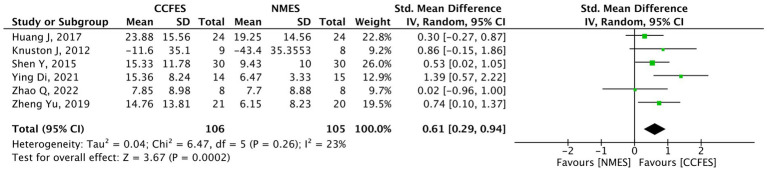
Forest plot of active range of motion (AROM). CCFES, contralaterally controlled functional electrical stimulation; NMES, neuromuscular electrical stimulation; SD, standard deviation; CI, confidence interval; Std. mean difference, standardized mean difference.

Pertaining to the 14 studies that assessed the upper limbs, the AROM was measured in eight studies. However, the analysis was only carried out on six (30, 33, 34, 38, 39, 41) as the other two (37, 44) were excluded from the analysis and are narratively described. The six studies’ pooled analysis show significant improvements favoring the intervention (SMD = 0.61, 95% CI: 0.29, 0.94, *p*-value = 0.0002, *I*^2^ = 23%, GRADE: moderate). The AROM’s forest plot can be seen in Figure 5.

Kim et al. (44) compared biofeedback functional electrical stimulation (BF-FES) with mirror therapy to functional electrical stimulation (FES) with mirror therapy and conventional physical therapy. They report that when it comes to the range of motion, a statistical significance favoring the BF-FES group was noted only in the wrist extension (*p*-value = 0.012) but not in the wrist flexion (*p*-value = 0.100) and elbow extension (*p*-value = 0.102). On the other hand, Zhou et al. (37) found that the AROM for shoulder flexion and abduction was statistically significant in the intervention group compared to the control (*p*-value <0.05).

#### 3.4.4 Box and blocks test

**Figure 6:**
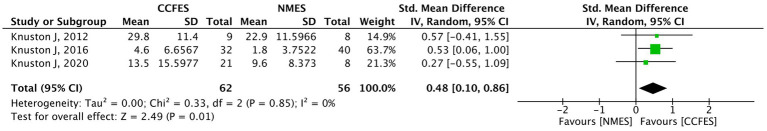
Forest plot of box and blocks test (BBT). CCFES, contralaterally controlled functional electrical stimulation; NMES, neuromuscular electrical stimulation; SD, standard deviation; CI, confidence interval; Std. mean difference, standardized mean difference.

BBT was assessed in four studies (30–32, 44). Kim et al. (44) were excluded from the pooled analysis due to the aforementioned reasons, and the study findings are narratively described. As a result, a total of 118 participants from three studies were incorporated into the quantitative synthesis. The meta-analysis shows a notable improvement in BBT favoring the CCFES groups (SMD = 0.48, 95% CI: 0.10, 0.86, *p*-value = 0.01, *I*^2^ = 0%, GRADE: very low) (Figure 6).

Kim et al. (44) found that BBT was enhanced in the BF-FES group by a 1.90 factor compared to baseline, which favors the intervention over the other two groups (*p*-value = 0.08).

#### 3.4.5 Arm motor ability test

**Figure 7:**
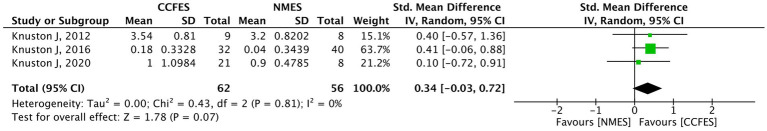
Forest plot of arm motor ability test (AMAT). CCFES, contralaterally controlled functional electrical stimulation; NMES, neuromuscular electrical stimulation; SD, standard deviation; CI, confidence interval; Std. mean difference, standardized mean difference.

Only three studies (30–32) (*N* = 118) measured the effect of the interventions on AMAT. The pooled analysis of these studies indicates statistical insignificance (SMD = 0.34, 95% CI: −0.03, 0.72, *p*-value = 0.07, *I*^2^ = 0%, GRADE: very low) (Figure 7).

#### 3.4.6 Modified Barthel index

**Figure 8:**
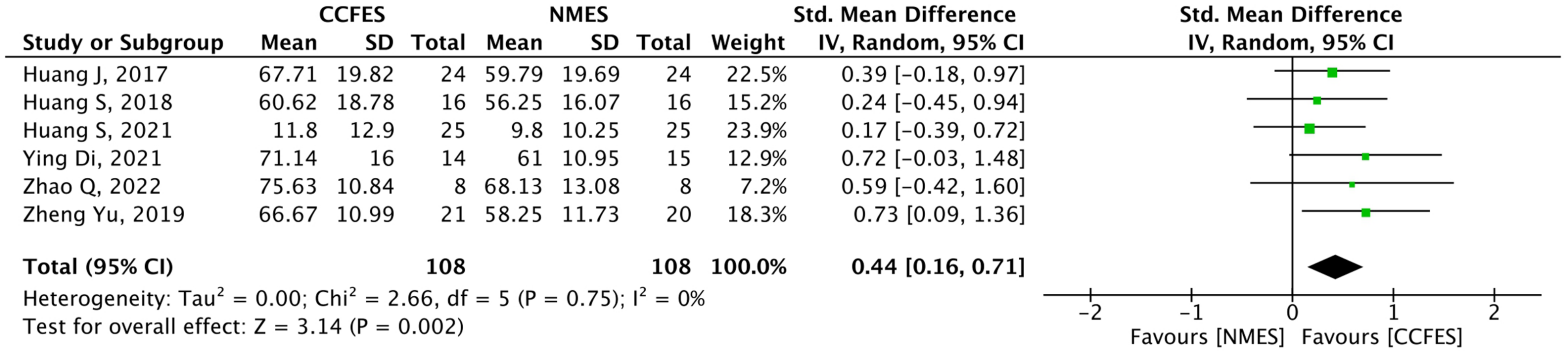
Forest plot of modified Barthel index (mBI). CCFES, contralaterally controlled functional electrical stimulation; NMES, neuromuscular electrical stimulation; SD, standard deviation; CI, confidence interval; Std. mean difference, standardized mean difference.

Seven studies (34–36, 38, 40–42) evaluated mBI. Shen et al. (42) compared the effect of the interventions in the lower limb, and thus, it was ruled out from the analysis. The meta-analysis on the remaining six studies reveal a significant upswing in favor of the CCFES group (SMD = 0.44, 95% CI: 0.16, 0.71, *p*-value = 0.002, *I*^2^ = 0%, GRADE: moderate) (Figure 8).

In Shen et al. (42), a strong improvement was seen in the CCFES group compared to the conventional NMES group (difference = 6.10, *p*-value = 0.024).

#### 3.4.7 Action research arm test

**Figure 9:**
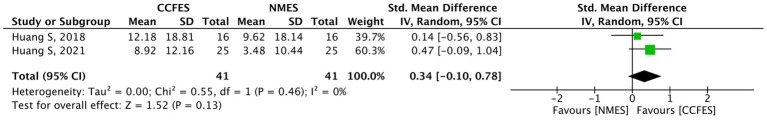
Forest plot of action research arm test (ARAT). CCFES, contralaterally controlled functional electrical stimulation; NMES, neuromuscular electrical stimulation; SD, standard deviation; CI, confidence interval; Std. mean difference, standardized mean difference.

ARAT was measured in two studies (35, 36). The results of the pooled analysis do not show any significant improvements between the interventions (SMD = 0.34, 95% CI: −0.10, 0.78, *p*-value = 0.13, *I*^2^ = 0%). The forest plot is shown in Figure 9. The outcome was assessed to have a low grade of evidence.

#### 3.4.8 Surface electromyography

**Figure 10:**
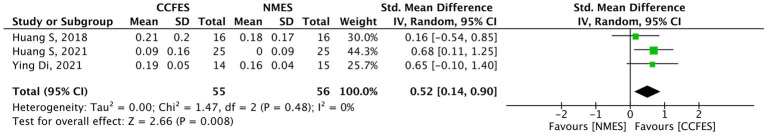
Forest plot of surface electromyography (sEMG). CCFES, contralaterally controlled functional electrical stimulation; NMES, neuromuscular electrical stimulation; SD, standard deviation; CI, confidence interval; Std. mean difference, standardized mean difference.

Four out of the included studies evaluated the sEMG results, of which only three (35, 36, 41) (*N* = 111) were involved in the pooled analysis. The analysis of the included studies reveals a significantly better level of improvement following the intervention in the CCFES group (SMD = 0.52, 95% CI: 0.14, 0.90, *p*-value = 0.008, *I*^2^ = 0%, GRADE: low) (Figure 10).

Shen et al. (42) again display that the CCFES group results were significantly higher than those of the conventional NMES group (increase = 16.93, *p*-value = 0.014).

## 4 Discussion

CCFES is a promising novel rehabilitation technique for limb paresis following a stroke. Inputs are derived from the volitional movements of the nonparetic limb, generating an equivalent stimulation in the affected limb (10). In this review, we conducted a systematic review and meta-analysis to compare the efficacy of CCFES and conventional NMES for limb hemiparesis following a stroke. Sixteen studies met the inclusion criteria, and 14 of them were included in the quantitative analysis. The results of the meta-analysis show that CCFES had a greater improvement in the following outcomes: UEFMA, BBT, mBI, AROM, and sEMG. However, the LEFMA, AMAT, and ARAT scores show no significant differences.

Upper limb impairment following a stroke is relatively common and occurs as a result of three main functional implications. Firstly, “a learned nonuse” in which muscular weakness prevents patients from using their affected limb. As a result, the patient may continue to not use their limb even after the passage of sufficient time, leading to the development of habitual behavior of immobility (5). Secondly, “a learned bad use” can develop when the injury prevents the normal movement of the upper limb, resulting in a compensatory mechanism to fulfill a certain task. This mechanism initially leads to success but are hard to maintain even with continual training, and with time it will eventually lead to a decline in performance (5). Finally, “forgetting” in which motor skills may not be retained due to breaks in rehabilitation or lack of variability in training (5, 47). Fortunately, the brain is inherently modifiable and demonstrates a high degree of neuronal plasticity. Mechanisms explaining the brain’s plasticity are still not fully understood. Physiological recovery of hemiparesis following a stroke allows for remodeling and recruitment of different areas in the brain. Within days after a stroke, distant connected cortical areas express an increased functional activity for some period and then decline in subsequent months. Furthermore, a reduction in the lateralized activation suggests a shift toward the unaffected hemisphere, which is evidenced by an increase in activity in the contralesional hemisphere. This reflects a reduced interhemispheric inhibition (IHI) following a stroke (48). Electrical stimulation therapies allow for similar patterns to occur by altering the inhibitory circuits and inducing long-term potentiation (49). A study by Cunningham et al. (15) found that CCFES therapy significantly reduced IHI when compared to cyclic NMES. Additionally, ipsilateral output was maintained following CCFES but was reduced after cyclic NMES. They reason that since CCFES therapy provides bilateral movements, it induces disinhibition which helps overcome fatigue-related diminution of the ipsilateral output.

The results of UEFMA in our study demonstrate that CCFES improves upper limb function significantly over conventional NMES. Although five of the analyzed studies showed a high risk of bias, a sensitivity analysis excluding the data of these studies was performed, and the attained results still favor the CCFES group. Furthermore, a subgroup analysis comparing the effects of different signal sensors elicit that an EMG sensor provides better improvements over a bend angle sensor. Although previous studies have indicated that both methods are effective and reliable (50), our findings convey a discrepancy between the two methods. However, the reasons for these differences could be due to the following three reasons: Firstly, two of the studies in the bend angle sensor group demonstrated a high risk of bias which questions the validity of their results and, thus, could have influenced the result of the meta-analysis. Secondly, an EMGB technique used in two studies in the EMG sensor group depicts an advantage over joint angle detection. Since EMGB contains a multi-channel detecting circuit, it allows for multiple movement training instead of a single movement offered by joint bend angle sensors (12). Lastly, two studies in the bend angle sensor group experimented on patients in the chronic phase of stroke. Studies suggests that recovery during the acute/subacute phases of stroke transpires more efficiently compared to the chronic phase. Dimyan and Cohen (51) have noted that the greatest influence on the motor cortex circuitry occurs within the first 3 months after a stroke. This suggests that the earlier the therapies are initiated after the incidence of a stroke, the more efficient the recovery will be. A previous meta-analysis of 36 clinical trials by Ottenbacher and Jannell (52) found that improvements in performance occurred as a result of early initiation of therapies, not the duration of such interventions. A similar pattern is noted in the LEFMA results. Despite there being only two studies comparing the effects of CCFES and conventional NMES in the lower limbs, the study by Shen et al. (42) analyzed patients in the subacute phase of stroke and illustrates a statistical significance in favor of the CCFES group. Knutson et al. (43) on the other hand, performed their trial on chronic stroke patients and portrayed a statistically insignificant result between the two groups. Another subgroup was performed in order to analyze the impact of CCFES when compared to conventional NMES regarding the long-term effects of these therapies. While we found that all of the end-of-treatment assessment was significant for CCFES, the subsequent follow-up assessment results contrasts the initial findings. Subsequent assessments at 1 month, 3 months, and 6 months all show no statistical significance between the two groups. These results conflict with what Lin et al. (53) study proclaims. In their study, the impact of 3 weeks of neuromuscular stimulation lasted for at least 6 months when compared to control subjects (53). One reason why our findings disaccord could be due to there being only a few studies that have truly investigated the long-term effects of CCFES therapy. As a result, the power of the evidence is not strong due to the limited number of studies. Additionally, most studies that have assessed patients at different follow-up periods have a high risk of bias, as well as have conducted their trials on chronic patients, which could have influenced the findings as previously discussed.

Other outcome measures investigated in this review include AROM, mBI, sEMG and BBT. Our results show that all four outcome measures favored CCFES therapy over conventional NMES. Even with the addition of several new RCTs (2 in AROM and 4 in mBI), the findings our analysis provides still support what Loh et al. (16) have found in their study. The additional effectiveness CCFES displays could be attributed to many reasons. One contributor to these advantages could be accredited to the interlimb coupling theory, where muscle groups from both sides of the body act as a single coordinated unit (54). Evidence that supports this theory was described in Cohen (55). In their experiment, alteration in the movements of the ipsilateral upper extremity occurred soon after movements were initiated from the contralateral upper limb. The modifications noted were either a halt, an increase or decrease, or a reversal of the direction of the movements being executed in the ipsilateral arm. They propose that the reason for these observed changes is due to an interference with the generation of motor commands in the brain (55). Since CCFES therapy is bilateral in nature and conventional NMES is unilateral, their corresponding neurophysiological mechanisms of recovery are expected to differ. Following a brain injury, cortical motor and sensory neuronal reorganization ensue (48). As a result of these reorganizations, different cortical circuits might become disinhibited, facilitating cortical plasticity and, thus, motor recovery (56). Several explanations for the observed neuronal reorganization were described by Donoghue et al. (57). One hypothesis they described is that changes in the efficacy of weakly stimulated pre-existing synapses and pathways allow for disinhibition to occur. Stinear and Byblow (58) explored the effect of rhythmical bilateral movements on disinhibition. They found that asynchronous upper limb movement maintained intracortical inhibition but was reduced during synchronous movements. This suggests that during bilateral synchronous movements, the unaffected hemisphere allows the damaged hemisphere to be reorganized (59). These principles, however, do not apply to unilateral and asynchronous bilateral movements (59). A previous systematic review and meta-analysis exploring the effect of bilateral arm training for post-stroke rehabilitation found that bilateral therapy alone or in combination with auxiliary sensory feedback, improved motor function in chronic and subacute stroke survivors (60).

Despite showing positive trends for CCFES, the results for the ARAT and AMAT were not significant statistically. Out of the 14 studies included in the quantitative analysis, ARAT was measured by only two studies and AMAT by three. The lack of high-quality RCTs assessing the effects of electrical rehabilitation therapy using these outcome measures and the low certainty of evidence these measures currently display could be a reason why a definitive conclusion was not reached. High-quality RCTs with a large sample size are still needed. A promising new multi-central clinical trial is currently being conducted by Knutson et al. (6), attempting to assess the effects of CCFES and conventional NMES on up to 129 patients. The results their study will provide will be quite imperative since it has a larger sample size and a long follow-up duration. Additionally, having the study be conducted at different sites will help confirm the results of previous trials. Future research on this topic should focus on incorporating additional outcome measures in their research as well as attempt to describe the effects of rehabilitation therapies on the different phases of stroke and assess the long-term effect of these therapies by following up with patients for long durations.

## 5 Strengths and limitations

There are several strengths that our review provides. Firstly, 10 new RCTs were incorporated in this study in addition to the previously reviewed six in Loh et al. (16). As a result, we believe that this adds to the power of the overall evidence since the results are updated and more inclusive. Secondly, several factors which were not explored previously have been analyzed in this review. These include LEFMA, sEMG, a comparison between different sensor modalities, and a long-term assessment of the efficacy of the interventions. Thirdly, all except one outcome displayed significant heterogeneity evident by the *I*^2^ test results, which validities and adds credibility to the presented data. Lastly, our research could serve as a guidance for future studies and guidelines regarding the applicability of CCFES for limb hemiparesis following a stroke.

We acknowledge that there are several limitations to our study. Firstly, some of the outcomes evaluated were analyzed by only two or three studies which limit their evidence as depicted by the GRADE score. Furthermore, despite the inclusion of 16 RCTs, an assessment of the efficacy of the interventions on the different phases of stroke could not be performed due to the limited data. Additionally, only one study compared CCFES to non-electrical stimulation therapy, thus, limited evidence exists regarding the effects of CCFES compared to non-electrical stimulation therapies. Lastly, only five studies were judged to have a low risk of bias. The lack of low risk RCTs impairs the certainty of the evidence presented.

## 6 Conclusion and implication on practice

This systematic review and meta-analysis add to the existing evidence regarding the benefits of CCFES for limb paresis following a stroke. CCFES showed better improvements in UEFMA, BBT, mBI, AROM, and sEMG scores when compared to the unilateral NMES. While in the other outcomes, the results were indifferent between the two groups which could have been due to the limitations mentioned above. Future studies should aim to have a higher quality methodology, and a proper sample size to increase the power of the findings. Furthermore, the effects of these therapies should be investigated on different parameters like the phase of stroke, lower limbs, duration of the intervention, and a long-term follow-up period.

## Data availability statement

The original contributions presented in the study are included in the article/Supplementary material, further inquiries can be directed to the corresponding author.

## Author contributions

AH: Conceptualization, Data curation, Formal analysis, Investigation, Methodology, Software, Validation, Writing – original draft, Writing – review & editing. AA: Conceptualization, Investigation, Methodology, Writing – original draft, Writing – review & editing. DB: Data curation, Writing – review & editing. RM: Data curation, Writing – review & editing. SA: Writing – review & editing. SM: Writing – review & editing.

## Footnotes

^1^ www.clinicaltrials.gov
